# Prevalence of Human Bocavirus in Africa and Other Developing Countries between 2005 and 2016: A Potential Emerging Viral Pathogen for Diarrhea

**DOI:** 10.1155/2018/7875482

**Published:** 2018-09-12

**Authors:** Mpumelelo Casper Rikhotso, Jean Pierre Kabue, Solanka Ellen Ledwaba, Afsatou Ndama Traoré, Natasha Potgieter

**Affiliations:** ^1^Department of Microbiology, School of Mathematical and Natural Science, University of Venda, Thohoyandou, South Africa; ^2^School of Mathematical Sciences, University of Venda, Thohoyandou, South Africa

## Abstract

**Background:**

Human Bocavirus (HBoV) is an emerging virus discovered in 2005 from individuals suffering gastroenteritis and respiratory tract infections. Numerous studies related to the epidemiology and pathogenesis of HBoV have been conducted worldwide. This review reports on HBoV studies in individuals with acute gastroenteritis, with and without respiratory tract infections in Africa between 2005 and 2016.

**Material and Method:**

The search engines of PubMed, Google Scholar, and Embase database for published articles of HBoV were used to obtain data between 2005 and 2016. The search words included were as follows: studies performed in Africa or/other developing countries or/worldwide; studies for the detection of HBoV in patients with/without diarrhea and respiratory tract infection; studies using standardized laboratory techniques for detection.

**Results:**

The search yielded a total of 756 publications with 70 studies meeting the inclusion criteria. Studies included children and individuals of all age groups. HBoV prevalence in Africa was 13% in individuals suffering gastroenteritis with/without respiratory tract infection.

**Conclusion:**

Reports suggest that HBoV infections are increasingly being recognized worldwide. Therefore, surveillance of individuals suffering from infections in Africa is required to monitor the prevalence of HBoV and help understand the role of HBoV in individuals suffering from gastroenteritis with/without respiratory tract infection.

## 1. Introduction

Diarrhea is a leading cause of morbidity and mortality in children worldwide [1, 2]. Diarrhea is the third major cause of childhood mortality in children less than 5 years of age especially in Africa and developing countries [3–5]. The modes of transmission include ingestion of contaminated food or water (e.g., via flies, inadequate sanitation facilities, sewage and water treatment systems, and cleaning food with contaminated water), direct contact with infected feces (fecal-oral route), person-to-person contact, and poor personal hygiene [6, 7].

According to WHO [1], approximately 90% of the estimated 2.2 million of deaths caused by diarrheal infections in children less than 5 years of age are related to poor sanitation and hygiene behaviors worldwide. While the mortality due to diarrheal diseases has declined significantly in children over the past twenty years in developed countries [8, 9], the incidence of childhood diarrhea in developing countries has not decreased [1, 2]. Those who survive these illnesses have repeated infections by enteric pathogens which remains a critical factor leading to serious lifelong health consequences [10] and eventually result in death [11]. Viruses are recognized as major cause of gastroenteritis, particularly in children, and the number of viral agents associated with diarrheal disease in humans has increased progressively. Viruses such as rotavirus, norovirus, astrovirus, and adenovirus that cause diarrhea have been reported worldwide [11].

The Human Bocavirus (HBoV) is a viral agent that has been reported worldwide in various studies as a potential cause of diarrhea outbreaks [5, 12–18]. The HBoV is a member of the Parvoviridae family, Parvovirinae subfamily, and the genus of* Bocavirus* [19–21]. The family Parvoviridae includes small, nonenveloped, icosahedral viruses with 5.3 kb single stranded DNA genome containing three open reading frames (ORFs); the first ORF, at the 5′ end, encodes NS1, a nonstructural protein [22]. The second ORF encodes NP1, a second nonstructural protein. The third ORF, at the 3′ end, encodes the two structural capsid viral proteins (VPs), VP1 and VP2 [23, 24]. There are currently four Bocavirus species identified worldwide, namely, HBoV1, HBoV2, HboV3, and HBoV4 [5, 25–28]. HBoV was first discovered in 2005 in children with acute respiratory tract infection [28]. In 2007, HBoV was detected in children suffering from gastroenteritis with and without symptoms of respiratory tract infections [14, 29–31]. Primary infection with HBoV occurs early in life in children between 6–24 months of age [32–34]; however, older children and adults can also be infected [28, 35]. Currently there is no specific approved treatment or vaccine for HBoV infection [25, 36, 37]. Since its discovery, the virus was mainly associated with respiratory tract infections, but recent studies have revealed the involvement of the virus in gastroenteritis. These studies indicate that only HBoV2, HBoV3, and HBoV4 strains of the virus are mainly involved in gastroenteritis [37–39]. Currently, there is limited data on ELISA method for the detection of the virus. HBoV detection has been done by conventional PCR [17, 28, 40, 41] and real-time PCR [42–45]. While HBoV epidemiological studies have shown evidence for widespread exposure to the virus, the causative role of HBoV in respiratory tract disease and gastroenteritis is still under investigation [46]. Proven evidence is difficult to obtain without an* in vitro* culture system and animal model [28, 47–49].

The prevalence of HBoV has been reported in Europe [50, 51], America [17, 41], Asia [34, 52], Australia [40, 53], Africa [54], and the Middle East [18], ranging from 1.5% to 19.3% [17, 55]. This review is a summary of reported HBoV studies in individuals with acute gastroenteritis, with and without respiratory tract infections looking specifically to studies in Africa to determine the role of HBoV in diarrheal outbreaks.

## 2. Materials and Methods

### 2.1. Search Strategy

**Figure 1:**
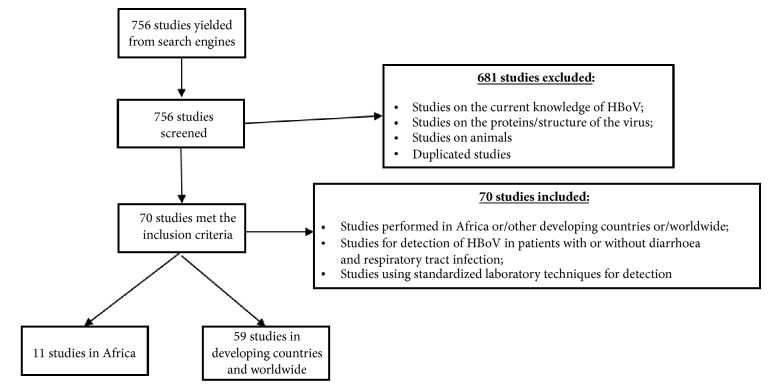
Schematic presentation of search engine used.

A literature search of selected studies that investigated HBoV in Africa, in other developing countries and worldwide was performed using the following terms: Human + Bocavirus + Africa + Developing countries + Worldwide on PubMed, Google scholar and Embase. This search yielded 756 publications (Figure 1). The first search was performed for HBoV + Africa, the second search was HBoV + other developing countries, and the third search was HBoV + worldwide. Keywords used included Human Bocavirus, Bocavirus, and Human parvovirus combined for each (Africa; Developing country; Worldwide). To avoid leaving out any studies not found in major scientific databases, Google search was also used. After reviewing each article, studies were selected if they met the following inclusion criteria:

(i) Studies performed in Africa/other developing countries/worldwide between 2005 and 2016.
(ii) Studies for the detection of HBoV in patients with or without diarrhea and respiratory tract symptoms. Diarrhea defined as the passage of loose or watery stools, at least three times in a 24-h period [56].
(iii) Studies using standardized laboratory techniques for detection of HBoV including PCR, real-time-PCR, and Multiplex PCR (m-PCR).

## 3. Data Extraction

Information extracted from the inclusion studies included country where study was done, time period of study, age range of participants, study setting (rural/urban/periurban), sampled population (number of included samples), method used for detection, clinical symptoms, sample type, and HBoV subtype.

## 4. Statistical Analysis

All analysis were conducted using R programming environment for data analysis and graphics Version 3.5.0 [57] to calculate random and fixed effects. Function “rma” from the package Metafor [58] was used to calculate heterogeneity between studies and generate a forest plot. Heterogeneity was assessed by Cochran's Q test.

## 5. Results and Discussion

Between 2005 and 2016 a total of 756 studies were published in Africa, other developing countries and worldwide. From these studies, 70 studies met the inclusion criteria of which 11 studies were from African countries and 59 studies combined were for other developing countries and worldwide. None of the studies reported on outbreaks (Tables 1, 2, and 3).

All 70 studies were reports on children ≤5 years of age (33%; 23/70) and children and individuals of all ages, ≥5 years (67%; 47/70). The majority of the studies (78%; 55/70) were reports on patients suffering from respiratory tract infection and 21.4% (15/70) were reports on patients suffering from diarrheal disease. Fifty-four studies (77%; 54/70) were done in urban settings and 23% (16/70) were done in rural settings (Table 1). The most reported HBoV subtype was HBoV1 (100%; 70/70), followed by HBoV2 (16%; 11/70), HBoV3 (13%; 9/70), and HBoV4 (7%; 5/70) (Table 1). A total of 54% (36/67) studies were done on samples collected from nasal swabs, 7% (5/67) were done on samples collected from throat swabs, 22% (15/67) were done on stool samples, 4% (3/67) were combined nasal/throat samples, 3% (2/67) were combined stool/nasal samples, 6% (4/67) were combination of nasal/stool/serum samples, and 3% (3/67) were a combination of nasal/serum samples.

**Figure 2:**
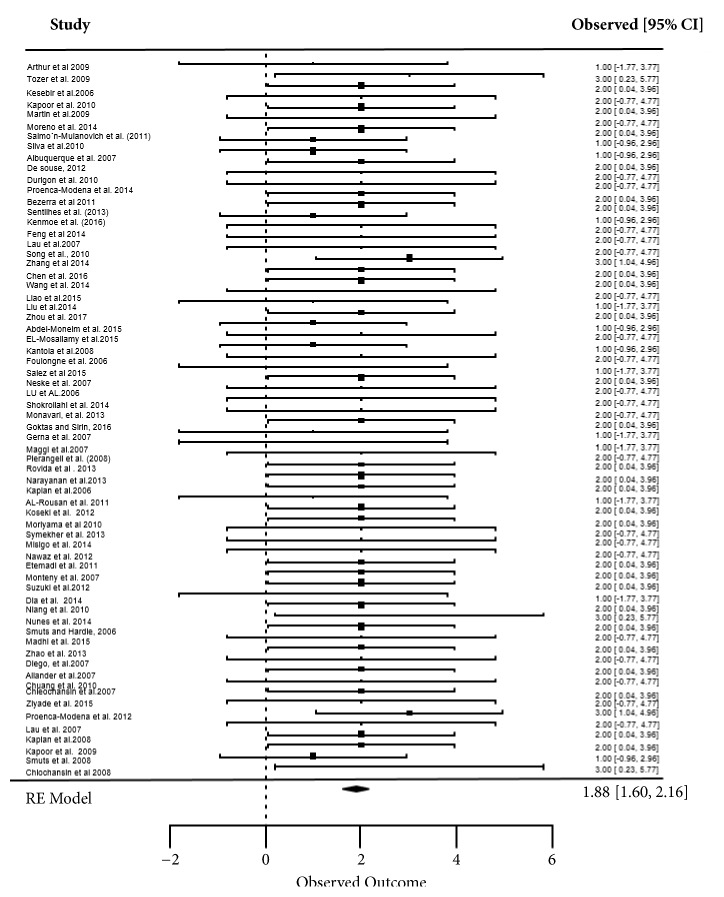
Forest plot for prevalence studies in detection of Human Bocavirus.

Meta-analysis was done to provide transparent, objective, and replicable summaries of the study findings. From all the 70 studies, 66 had sufficient information to enable statistical analysis. As shown in Figure 2 with the dispersion in study prevalence, there was a low heterogeneity among the studies (Cochran Q = 12.2800 [df = 65] P-Val = 1). Apart from the observed increase in the prevalence of HBoV, none of the other drivers (including age, setting, symptoms, method of detection, and hospitalization) achieved statistical significance. The test for overall effect was Z = 13.29 (P<0.0001) which was highly significant in the findings.

Ten studies were from other developing countries of which eight studies (80%) reported on patients suffering from respiratory tract infection and two studies (20%) reported on patients suffering from diarrheal disease. All ten of the studies focused only on children (100%; 10/10) (Table 2). All studies in other developing countries worked on hospitalized patients (Table 2). A total of 70% (7/10) of the studies collected nasal swabs, 20% collected throat swabs, and 20% of the studies collected stool samples. The most sampled population was children ≤ 6 years of age (Table 2). Majority 60% (6/10) were done in urban setting in other developing countries while 40% (4/10) were done in rural settings. Eight (80%) of the studies reported on patients suffering from respiratory tract infections (Table 2). In other developing countries, HBoV was reported in Argentina 10% (1/10), Cambodia 10% (1/10), China 40% (4/10), India 10% (1/10), Jordan 10%(1/10), and the Philippines 10%(1/10).

In Africa, the majority of studies (82%; 9/11) were done in urban settings while 18% (2/11) were done in rural settings. Ten (91%) of the studies reported on patients suffering from respiratory tract infections and one study (9%) reported on patients suffering from gastroenteritis. In these studies, a total of 383 (10.4%) samples tested positive for HBoV (Table 3). More studies reported HBoV in children less than five years of age (54%; 6/11) compared to children above the age of 5 and adults 45% (5/11) (Table 3).

Countries that reported on HBoV in Africa included Kenya 18% (2/11), South Africa 36% (4/11), Egypt 18% (2/11), Cameroon 9% (1/11), and Senegal 18% (2/11). Five of the 11 studies in Africa focused on hospitalized patients and 36% (4/11) studies focused on outpatients, while 18% (2/11) studies focused on both hospitalized and outpatients. Eight (73%; 8/11) of the studies in Africa collected nasal swabs, two studies 18% (2/11) collected throat swabs, and one (9%; 1/11) study collected stool samples.

The prevalence of HBoV in Africa was 13% in individuals suffering from gastroenteritis with and without respiratory tract symptoms. The high detection rate of HBoV in Africa was consistent with the global increase of HBoV in children less than 5 years of age [4, 59]. Children of all age group are most likely to experience HBoV infection as a result of poor sanitation and hygiene practices [59]. The most predominant HBoV subtype identified in Africa was HBoV1, which was detected in all the studies. Only one study (9%), from Kenya detected all subtypes (HBoV1-4) from children of all age group (Table 3). Not all the studies tested for all HBoV subtypes; this may be due to the fact that other HBoV subtypes have just been recently discovered compared to HBoV1 [37]. The most predominant HBoV subtype identified in other developing countries was HBoV1, which was isolated in all the studies. Only 10% (1/10) of the studies from china detected HBoV2 in the study population (Table 2).

The results in Africa indicated that HBoV in children less than five years of age was high, 54% (6/11) compared to children above 5 years of age and adults 45% (5/11) (Table 3). Schildgen and colleagues [60] showed that all age groups can be affected by HBoV, although severe infections requiring hospitalization occur primarily in patients with an underlying disease and children under 5 years of age [61–64]. Severe clinical cases (such as destruction of the epithelium of the respiratory system) have been described in children [61, 64–66] and adults with immunodeficiency [64] and other risk groups [67]. Studies in Africa (13%; 9/70) were mostly done in urban setting compared to other developing countries/worldwide (87%; 61/70) (Tables 1, 2, and 3). This could be due to the lack of laboratory resource capacity and technology for the detection of HBoV in rural settings.

The methods used for detecting HBoV have been conventional PCR [17, 28, 40, 41, 45, 53, 64] and real-time PCR [42–45, 54, 68], due to the limited success of serological and viral culture techniques. Real-time PCR is more sensitive and offers greater sensitivity, increased specificity with the addition of oligoprobes, and the added benefit of a closed detection system, reducing the likelihood of false positive results due to contamination with amplicon [33]. In Africa, 63% (7/11) of studies used conventional PCR for detection, 27% (3/11) used real-time PCR, and 9% (1/11) used Multiplex PCR which is also conventional PCR (Table 3).

In all eleven African studies, HBoV1 was detected (Table 3), similarly in other developing countries HBoV1 was detected in all the studies. HBoV Subtype 1 is mainly associated with respiratory diseases but can also be found in stool samples from patients suffering from diarrhea. Previous studies have reported prevalence of HBoV in symptomatic patients 1.5–16% worldwide [69, 70]. Several studies have isolated HBoV from children with respiratory tract infection worldwide, and the prevalence of HBoV in these children was 1.5%–19% [32, 33, 35].

The reports on HBoV in Africa, other developing countries, and worldwide in individuals suffering from respiratory tract infection 78% (55/70) were higher compared to those suffering from gastroenteritis 21% (15/70) (Tables 1, 2, and 3). This could be due to the fact that most studies focused on HBoV in respiratory tract infection since its discovery in 2005 [28, 33, 36, 71]. However recent studies are increasingly detecting HBoV in individuals suffering from diarrheal diseases due to the presence of the virus in stool samples of individuals suffering from gastroenteritis [37, 72–74].

Although the number of studies in Africa is limited, the HBoV prevalence rate of 13% indicates that this virus is one of the emerging viral agents in those suffering from diarrhea with and without respiratory tract infections. Currently there is no available reporting system for HBoV infection in the primary healthcare systems in Africa, suggesting that diarrheal cases with and without respiratory tract infection are likely to be underreported [47]. The high frequency of HBoV in children raises a potential health risk as these children may act as reservoir for other emerging epidemic HBoV strains [27, 37, 75].

## 6. Conclusion

More studies are required in Africa, especially in rural settings to monitor the prevalence of HBoV and help understand the role of HBoV in individuals suffering from gastroenteritis with/without respiratory tract infection. HBoV infections are likely to be underreported in Africa considering the costs of testing for the virus. This review was done to shed light on HBoV and its possible role in diarrheal incidence.

## Figures and Tables

**Table 1:** Human Bocavirus globally, studies published between 2005 and 2016.

**Country**	**Study period**	**Setting**	**Age range**	**Sampled population**	**Tested samples**	**Positive samples (**%**)**	**Hospitalized**	**Outpatient**	**Sample type**	**Symptoms**	**Detection method**	**HBoV type**	**Reference**
Australia	2001	-	-	children	197	125 (63.5%)	197	-	Stool	Diarrhea	Nested PCR	1,2,3	[25]
	2003-2004	Urban	≤ 5 years	children	700	41 (6%)	604	96	Nasal–throat, stool, whole blood	Respiratory infection/diarrhea	Real-time PCR	1	[26]

America	2004	Urban	< 2 years	children	1271	22 (1.7%)	1271	-	Nasal	Respiratory tract infection	PCR	1	[41]
	-	Peri-urban	All	children	641	101 (16%)	-	-	Stool	Diarrhea	PCR	1,2,3,4	[74]
	2007-2008		2-11 years	children	149	7 (5%)	-	149	Throat, Nasal	Respiratory tract infection	Real-time PCR	1	[66]

Argentina	2011	Peri-urban	≤ 2 years	children	222	15 (7%)	222	-	Nasal	Respiratory tract infection	PCR	1	[76]

Argentina, Nicaragua, Peru	-	-	< 6 years	children	568	61 (11%)	568	-	Nasal	Respiratory tract infection	Real time PCR	1	[77]

Brazil	2004-2007	Peri-urban	≤ 2 years	children	397	3 (0.76%)	-	397	Nasal, throat	Respiratory tract infection	PCR	1	[62]
	2003-2005	Peri-urban	<15 years	children	705	14 (2%)	285	420	Stool	Diarrhea	PCR	1	[15]
	1998-2004	Urban	< 5 years	children	762	44 (5.8%)	762	-	Stool	Diarrhea	PCR	1,3	[78]
	2008	Urban	<2 years	children	511	55 (11%)	511	-	Nasal	Respiratory tract infection	PCR	1,2,3	[79]
	2010-2012	Urban/rural	≤18	children	200	67 (33.9%)	200	-	Nasal	Respiratory tract infection	Real-time (RT-PCR)	1	[80]
	2010-2011	Urban/rural	1-14 years	children	121	36 (29.8%)	121	-	Nasal	Respiratory tract infection	Real-time PCR	1	[81]
	2005-2007	Urban/rural	All	Children/adults	1015	49 (4.8%)	1015	-	Nasal	Respiratory tract infection	PCR	1	[82]
	2008-2009	Urban	< 5 years	children	407	77 (19%)	407	-	Nasal	Respiratory tract infection	PCR	1	[83]
	2006-2007	Urban/rural	All	Children/adults	90	2 (2%)	90	-	Stool	Diarrhea	PCR	1	[84]

Cambodia	2009-2010	Urban	All	Children/adults	292	162 (55%)	292	-	Nasal, throat	Respiratory tract infection	Multiplex PCR	1	[85]

Cameroon	2011-2013	Urban	≤15 years	children	347	37 (11%)	347	-	Throat	Respiratory tract infection	Multiplex PCR	1	[86]

China	2009-2013	Urban/rural	All	Children/adults	29248	551 (2%)	29 248	-	Nasal	Respiratory tract infection	Real-time PCR	1	[87]
	2004-2005	Urban	< 18 years	children	203	83 (40%)	203	-	Stool, Nasal	Respiratory infection/diarrhea	PCR	1	[88]
	2007-2008	Urban	≤15 years	children	235	21 (9%)	235	-	Nasal	Respiratory tract infection	PCR	1,2	[89]
	2009-2012	Urban/rural	All	Children/adults	14237	180 (1.26%)	14237	-	Nasal	Respiratory tract infection	PCR	1	[23]
	2012-2013	Urban/rural	<14 years	children	4130	(16.7%)	4130	-	Throat	Respiratory tract infection	Real-time PCR	1	[90]
	2012	Peri-urban	≤ 5 years	children	122	-	-	122	Stool	Diarrhea	Multiplex real-time PCR	1	[91]
	2009-2014	-	All	Children/adults	12502	225 (2%)	12502	-	Nasal	Respiratory tract infection	PCR	1	[92]
	2009-2014	Urban	<14 years	children	4242	125(3%)	4242	-	Nasal	Respiratory tract infection	Real time PCR	1	[93]
	2012-2013	Urban	< 6 years	children	346	60 (17.34%)	346	-	Stool	Diarrhea	PCR	1,2	[94]

Egypt	2013-2014	Urban	≤ 36 months	children	95	54 (56.8%)	95	-	Nasal	Respiratory tract infection	Real-time PCR	1	[8]
	2013-2015	Urban	1 month-2 years	children	100	2(2%)	100	-	Stool	Diarrhea	PCR	1	[95]

Finland	2000-2002	Urban	3 months-15 years	children	117	24 (49%)	117	-	Nasal, serum	Respiratory tract infection	Qualitative PCR	1	[96]
	2010	Urban	All	Children/adults	250	4 (1.6%)	250	-	Stool	Diarrhea	Multiplex real-time quantitative PCR	1,2,3,4	[27]

France	2003-2004	-	< 5 years	children	589	9 (1.5%)	589	-	Nasal	Respiratory tract infection	PCR	1	[51]
	2010-2011	Urban	All	Children/adults	1465	5 (0.3%)	1465	-	Nasal	Respiratory tract infection	Multiplex PCR	1	[97]

Germany	2007	Urban	-	children	834	115 (14%)	834	-	Stool, nasal, serum	Respiratory tract infection	Real-time PCR	1	[98]

Hong Kong	2004-2005	Periurban	-	children	1178	12 (1%)	1178	-	Nasal	Respiratory tract infection	Real time PCR; PCR	1	[99]
	2004-2005	Urban	<18 years	children	3035	103 (3.4%)	3035	-	Nasal	Respiratory tract infection	PCR	1	[88]

Iran	2009-2011	Peri-urban	2-108 months old	children	80	6(8%)	80	-	Stool	Diarrhea	Real-time PCR	1	[100]
	2010-2011	Urban	<4 years	children	200	16 (8%)	200	-	Stool	Diarrhea	Real-time PCR	1	[101]

Istanbul	2014-2015	Urban/rural	All	Children/adults	845	91 (11%)	845	-	Nasal	Respiratory tract infection	Real time PCR	1	[102]

Italy	2005-2006	Urban	-	Children/adults	426	42 (9.9%)	426	-	Nasal	Respiratory tract infection	PCR	1	[103]
	2000-2006	Urban	All	Children/adults	355	4.5%,	355	-	Nasal	Respiratory tract infection	PCR	1	[30]
	2004-2007	Urban/rural	<14 years	children	415	34 (8.2%)	415	-	Nasal	Respiratory tract infection	PCR	1	[104]
	2011-2012	Urban	All	Children/adults	689	14 (2%)	689	-	Stool	Diarrhea	Real time PCR	1	[105]

India	2010-2011	Rural/Peri-urban	0–6 years	children	300	2 (0.67%)	300	-	Throat	Respiratory tract infection	PCR	1	[106]

Jordan	2003-2006	Peri-urban	< 5years	children	326	57 (17%)	326	-	Nasal	Respiratory tract infection	PCR	1	[18]
	2007	Peri-urban	≤13 years	children	220	20 (9%)	220	-	Nasal	Respiratory tract infection	PCR/ real-time PCR	1	[107]
	2003-2004	Urban	≤ 5 years	children	326	57 (17%)	326	-	Nasal	Respiratory tract infection	Real time PCR	1	[108]

Japan	2005-2011	Peri-urban	0-136 months	children	850	132 (15.5%)	850	-	Nasal	Respiratory tract infection	Nested PCR	1,2,3,4	[73]
	2007–2009	Urban/rural	<2 years	children	402	34 (8.5%)	402	-	Nasal	Respiratory tract infection	PCR	1	[109]

Kenya	2013	Rural	≤5 years.	children	125	21 (17%)	125	-	Throat	Respiratory tract infection	PCR	1	[59]
	2007-2009	Urban	All age group	Children/adults	384	7 (1.8%)	-	384	Nasal	Respiratory tract infection	PCR	1,2,3,4	[3]

United Kingdom	1993–1996	Urban/rural	All	Children/adults	4380	324 (7.4%)	4380	-	Stool	Diarrhea	Real time PCR	1,2,3	[110]

Malaysia	2012	Urban/rural	Children	children	1	1 (99%)	1	-	Nasal	Respiratory tract infection	PCR	1	[111]

Netherland	2005-2006	Peri-urban	3 months-6 years	children	257	4 (1.6%)	-	257	Nasal	Respiratory infection/diarrhea	Real time PCR	1	[112]

Pakistan	2008	Rural	-	Children/adults	98	-	-	-	Stool	Diarrhea	PCR	1,2	[113]

Philippines	2008-2009	Urban	8 days to 13 years	children	1242	2 (0.16%)	1242	-	Nasal	Respiratory tract infection	PCR	1	[114]

Senegal	2009-2011	Urban	All age group	Children/adults	232	1 (0.4%)	232	-	Nasal	Respiratory tract infection	Real-Time PCR	1	[115]
	2007	Rural	≤5	children	82	1 (1.2%)	82	-	Nasal	Respiratory tract infection	PCR	1	[4]

South Africa	1998-2000	Urban	<2	children	1460	332 (22.8%)	1460	-	Nasal	Respiratory tract infection	RT-PCR	1	[116]
	2004	Urban	2 days–12 years	children	341	13 (37%)	341	-	Nasal	Respiratory tract infection	PCR	1	[54]
	2004-2005	Urban	2 months to 6 years	children	242	18 (7.4%)	242	-	Nasal	Respiratory tract infection	Nested PCR	1	[117]
	2009-2010	Rural	3 months to <5 years	children	260	30 (11.5%)	-	260	Nasal	Respiratory tract infection	PCR	1	[118]

Shanghai	2009-2012	Peri-urban	≤ 5 years	children	554	39 (7.0%)	554	-	Nasal, stool, whole blood	Respiratory tract infection	Real time PCR/ PCR	1	[29]

Spain	2005-2006	Urban	<3 years	children	527	48 (9.1%)	527	-	Stool, Nasal	Respiratory tract infection/diarrhea	PCR	1	[14]

Sweden	2000-2002	Urban	3 months to 15 years	children	259	49 (19%)	259	-	Nasal, serum	Respiratory tract infection	Real-time PCR	1	[42]

Taiwan	2008-2009	Peri-urban	5 months-9 years	children	705	35 (5%)	-	705	Throat	Respiratory tract infection	PCR	1	[119]

Thailand	2006	Urban	1 month-9 years	children	252	18 (7%)	252	-	Nasal	Respiratory tract infection	PCR	1	[20]
	2005-2007	Peri-urban	2 months-5 years	children	427	2 (0.4%)	225	202	Stool	Diarrhea	PCR	1	[33]

Turkey	2015	Urban	Five months	children	1	1 (99%)	1	-	Nasal, stool	Respiratory tract infection/diarrhea	Multiplex PCR	1,2,3,4	[120]

**Table 2:** Human Bocavirus studies in other developing countries between 20005 and 2016.

**Country**	**Study period**	**Setting**	**Age range**	**Sampled population**	**Tested samples**	**Positive samples (**%**)**	**Hospitalized**	**Outpatient**	**Sample type**	**Symptoms**	**Detection method**	**HBoV type**	**Reference**
Argentina, Nicaragua and Peru	-	-	< 6 years	children	568	132 (23%)	568	-	Nasal	Respiratory tract infection	Real time PCR	1	[77]

Brazil	2007	Rural	<3 years	children	260	27 (10.4)	260	-	Nasal	Respiratory tract infection	Real-time PCR	1	[121]

Cambodia	2009-2010	-	All	Children/adults	292	9 (3%)	292	-	Throat swabs, nasal	Respiratory tract infection	Multiplex real-time PCR	1	[85]

China	2012	Peri-urban	≤ 5 years	children	122	-	-	-	Stool	Diarrhea	Multiplex real-time PCR	1	[91]
	2009-2014	-	All	Children/adults	12502	225 (2%)	12502	-	Nasal	Respiratory tract infection	PCR	1	[92]
	2009-2014	Urban	<14 years	children	4242	125 (3%)	4242	-	Nasal	Respiratory tract infection	Real time PCR	1	[98]
	2012-2013	Urban	< 6 years	children	346	60 (17.34%)	346	-	Stool	Diarrhea	PCR	1,2	[94]

India	2010-2011	Rural/Peri-urban	0–6 years	children	300	2 (0.6 %)	300	-	Throat swabs	Respiratory tract infection	PCR	1	[106]

Jordan	2003-2004	Urban	≤ 5 years	children	326	57 (17%)	326	-	Nasal	Respiratory tract infection	Real time PCR	1	[108]

Philippines	2008-2009	Urban	8 days to 13 years	children	1242	2 (0.16)	1242	-	Nasal	Respiratory tract infection	PCR	1	[114]

**Table 3:** Human Bocavirus studies in Africa between 2005 and 2016.

**Country**	**Study period**	**Setting**	**Age range**	**Sampled population**	**Tested samples**	**Positive Samples (**%**)**	**Hospitalized**	**Outpatient**	**Sample type**	**Symptoms**	**Detection method**	**HBoV type**	**Reference**
Cameroon	2011-2013	Urban	Children aged ≤15 years	children	347	37 (10.6%)	347	-	Throat	Respiratory tract infection	Multiplex PCR	1	[86]

Egypt	2013-2015	Urban	1 month-2 years	children	100	2 (2%)	40 (40%)	60 (60%)	Stool	Diarrhea	PCR	1	[95]
	2013-2014	Urban	≤ 36 months	children	95	54 (56%)	11 (40%)	43 (63%)	Nasal	Respiratory tract infection	Real-time PCR	1	[8]

Kenya	2013	Urban	≤5	children	125	21 (16.8%)	125	-	Throat	Respiratory tract infection	PCR	1	[59]
	2007-2009	Urban	All age group	Children/adults	384	7 (1.8%)	-	384	Nasal	Respiratory tract infection	PCR	1,2,3,4	[3]

Senegal	2007	Rural	≤5	children	82	1 (1.2%)	-	82	Nasal	Respiratory tract infection	PCR	1	[4]
	2009-2011	Urban	All age group	Children/adults	232	1 (0.43%)	-	232	Nasal	Respiratory tract infection	Real-Time PCR	1	[115]

South Africa	1998-2000	Urban	<2	children	1460	174 (22.8%)	1460	-	Nasal	Respiratory tract infection	RT-PCR	1	[116]
	2004	Urban	2 days–12 years	children	341	38 (11%)	341	-	Nasal	Respiratory tract infection	PCR	1	[54]
	2004-2005	Urban	2 months to 6 years	children	242	18 (7.4%)	242	-	Nasal	Respiratory tract infection	Nested PCR	1	[117]
	2009-2010	Rural	3 months to <5 years	children	260	30 (11.5%)	-	260	Nasal	Respiratory tract infection	PCR	1	[118]
